# Supplementary material for: “Support for my dad would have benefited me because I was the one looking after him”: A qualitative analysis of the support needs of young people exposed to Adverse Childhood Experiences
Source: PLoS One. 2025 Apr 22;20(4):e0313371. doi: 10.1371/journal.pone.0313371 (PMC12013905; doi:10.1371/journal.pone.0313371)
Supplement: S1 Table — (DOCX) [file pone.0313371.s001.docx]

# Supporting Information 1

#### S1 Table. Sample demographics from a survey of young people in Wales regarding their Adverse Childhood Experiences, organised to show those who left a comment

No comment Left a comment

|  |  | Number | % | Number | % |
| --- | --- | --- | --- | --- | --- |
| Gender* | Male | 89 | 21.2 | 38 | 27.3 |
|  | Female | 299 | 71.2 | 82 | 59.0 |
|  | Non-binary | 22 | 5.2 | 10 | 7.2 |
|  | Other | 10 | 2.4 | 8 | 5.8 |
| Age (years)* | 16 | 146 | 34.8 | 45 | 32.4 |
|  | 17 | 180 | 42.9 | 63 | 45.3 |
|  | 18 | 92 | 21.9 | 30 | 21.6 |
| ACEs | 1 ACE | 111 | 26.4 | 14 | 10.1 |
|  | 2-3 ACES | 139 | 33.1 | 55 | 39.6 |
|  | 4 or more ACEs | 170 | 40.5 | 70 | 50.4 |
