# Supplementary material for: “Support for my dad would have benefited me because I was the one looking after him”: A qualitative analysis of the support needs of young people exposed to Adverse Childhood Experiences
Source: PLoS One. 2025 Apr 22;20(4):e0313371. doi: 10.1371/journal.pone.0313371 (PMC12013905; doi:10.1371/journal.pone.0313371)
Supplement: Attachment — Submitted filename: SarahLester_reviewercomments.docx [file pone.0313371.s002.docx]

**“Support for my dad would have benefited me because I was the one looking after him”: A**

**qualitative analysis of the support needs of young people exposed to Adverse Childhood**

**Experiences**

Overall, the paper is comprehensive, well-written and makes an important contribution to the evidence base. You make a strong argument for why it’s important to understand young people’s experiences and views by way of a broader sample, rather than exclusively seeking the views of those who are already accessing services, and your methodology enables you to elicit views from a broader pool of experience accordingly. As you delineate, not only is it important to consider the social forces which stigma, shame and normalisation play within young people’s (non)disclosure of ACE experience, but it is also important to do so within the context of severe constraints on public sector funding which shapes young people’s (lack of) access to services and the quality of service provision.

The approach you have taken is particularly valuable for illuminating the interdependencies between young people and their family members (as demonstrated in Theme 1: Help me by helping my family), as well as the interconnectedness of ACE experiences (it is striking that 50.4% of respondents who left a comment had experience of 4 ACEs or more) and the interrelationships between young people’s ACE experience and mental health. This helps to surface some of the complexities of how young people experience ACEs in their life from their own, rather than pre-determined or clinical, perspectives.

**Overarching comments**

There were four overarching aspects of the paper which I felt would benefit from closer scrutiny or explanation. First, the inclusion of ‘long-term health diagnosis’ in your modified definition of ACEs, second, the gendered elements of your findings, third, the extent to which the free text responses were considered in the context of the other information respondents provided, and lastly, ethical considerations throughout the research process. I will take each of these in turn below.

1) **Inclusion of ‘long-term health diagnosis’** One point of clarification which would be useful in improving the paper is a more in-depth justification for your decision to modify the CDC’s Behavioural Risk Factor Surveillance System list of experiences by way of including 'long-term health diagnosis’. While you explain: “This measure was modified through co-production with young people’ there is no explanation provided for the rationale underlying this decision. While not wanting to diminish the value which public engagement/ co-production with young people had in shaping your work, it would be useful to hear more about the process through which this extra dimension was added For example, was the suggestion subjected to any kind of critical interrogation or considered in the context of the wider literature? What did the discussion leading to this decision comprise of?

On a practical level I was unclear whether ‘long-term health diagnosis’ referred to the young person’s direct experience, or related to living in a household where someone else had a diagnosis? Or whether it referred to both? Perhaps including the questionnaire as an appendix would help clarify matters (apologies if this info had been provided – additional materials did not appear to include this as far as I could tell).

Conceptually, if ‘long term health diagnosis’ referred to young people’s own diagnosis this would represent quite a shift from the original ACEs framework (Felitti, et al. 1998) which predominantly relates to issues experienced in households or through violence/trauma experienced as a result of others. While, as you say, ACEs affect health and well-being in various ways, including a health condition in this way appears to conflate pathways somewhat (although, arguably, as your work alludes to, the pathways have recursive or self-reinforcing elements to them). All the same, and given that 47.4% of respondents reported ‘health condition’ in their responses, it is important to provide clarification on this point.

2) **Gendered dimension** While it tapers off slightly once you are left with the percentage of females who provided free text responses (from 71.2% of respondents down to 59%), there is still a considerable ‘over-representation’ of female or non-binary respondents when compared to the composition of the wider population (latest census data suggests from England and Wales posits 0.06% identify as non-binary) which appears overlooked within the analysis/ discussion. It is also not clear why trans categories were not included. While non-disclosure, non-reporting, not accessing services renders it challenging to compare % gender categories to wider ACE prevalence figures, it would be useful to know the extent to which you were able to disaggregate (or not) data according to these categories. This is not to say it would have necessarily have been the appropriate approach, but more to raise that it would be useful to know how considerations around gender informed your processes.
3) **Contextualisation** Related to the above point (and, also, the methods provided for the qualitative analysis, as I go on to discuss below), it would elevate the paper if you provided more information on how free text responses were analysed in relation to the other information provided by the respondent, or not. Your description implies free text analysis took place in a decontexualised way (as in free text responses were analysed as a discrete set of data), but I may have misunderstood. Another consideration, which is not discussed, is the extent to which the questionnaire sought information on whether the young person had disclosed their ACE experience. Again, none of this is to say this would be the right approach or not, just that the information would help contextualise the findings. As already suggested – including the questionnaire in an appendix would help clarify this, without affecting word count. While you refer to the role of ‘normalisation’ in your discussion, there is perhaps more nuance that could be introduced in terms of the role of non-disclosure in terms of whether or not young people felt they had received enough support or not’. This appears especially relevant given the highest percentage category which reported not receiving enough support was for sexual abuse, where lags in time before disclosure tend to be much longer than for other ACEs.
4) **Ethical considerations** While it is commendable that you provide the following information: ‘The first author FA conducted the workshops, she is a female PhD student with experience of teaching 11-18 year olds. A relationship with participants was not established prior to study commencement.’ it does raise considerations both around the extent to which a one-off workshops could be considered as representing true ‘co-production’ (with parity of power between researcher and young people) and the safeguarding which was in place for potentially (re)traumatising discussions. This may be more of a semantic issue, as most of what you describe seems more closely aligned with public involvement in research (rather than co-production), and the role of including young people appears to be bring in extra perspective of those with relevant experience living in the same geographical area (although this is implicitly rather than explicitly stated). However, given that ‘co-production’ is such a contested term (see, for example, Oliver et al. 2019 and response from Williams et al. 2020- references below) , it is particularly important to consider the language which used around these processes and I would advise you to reflect on the extent to which you can justify using ‘co-production’ in this context. Reflecting on power dynamics specifically: ‘Participants completed discussion activities to ensure they understood the role of participants and the researcher as equal partners in co-production.’ this does imply a rather hierarchical one-way approach to ‘making it be understood’ as opposed to genuinely co-producing ground rules and generating an equal power dynamic with young people. There is little sense of how the process of involving young people affected the knowledge produced or what the researcher took away from the experience, which belies the two-way learning and equal power dynamic which ‘co-production’ implies. I appreciate this is challenging to address within the constraints of a single journal article, and perhaps warrants its own output, but some more reflexivity on the adequacy of the approach and how the young people’s involvement affected the process would be useful – as might some additional material (for the appendices) on recruitment and other communication around these activities which was presented to the young people involved.

Re: safeguarding: how were safeguarding issues considered across workshops and survey interactions with young people? Was it possible to signpost, offer support if required etc?

**Specific in-text comments**

Beyond these main points, I have a small number of comments relating to specific sections or statements made in the paper, which I take in order below:

**2.5 Qualitative Analysis**

This section reads in quite generic terms, and tends to veer towards describing Braun and Clarke’s stages of reflexive thematic analysis rather than giving any insights into your own specific processes or rationales. This was especially surprising given that you describe using ‘reflexive’ qualitative analysis. In relation to your use of ‘deductive’, you appear to use this as if relating to being structured towards your research question (which would be the case with any form of analysis) - if you mean ‘deductive’ in the sense of grounded in pre-existing theoretical concepts, that could be made clearer. As discussed above, under ethical

**4. Discussion “**Supportive responses to ACEs should support the whole family; for many young people, ACEs are not distinct, logical experiences but rather features of a complicated family system.”**.** Perhaps this is more of a personal take, but I’m not convinced of the use of the word ‘logical’ in the above sentence – mainly because it carries the inference that ACEs could be experienced as somehow ‘illogical’. Are you suggesting that ACEs are experienced in ‘emergent’ ways or as ebbs and flows – just wonder how else this might be expressed?

Just one last, very minor point. The following quote appears twice (Theme 1 and then later in Theme 4)

*“I never knew where or how to speak to people about my feelings; I bottled it all up and kept*

*them to myself.” (Participant 557)*

Is this intentionally repeated or should it only appear within one theme?

REFERENCES

Felitti, V. J., Anda, R. F., Nordenberg, D., Williamson, D. F., Spitz, A. M., Edwards, V., Koss, M. P., & Marks, J. S. (1998). Adverse Childhood Experiences Study Questionnaire [Database record]. PsycTESTS.

Oliver, K., Kothari, A. and Mays, N. (2019). ‘The dark side of coproduction: do the costs outweigh the benefits for health research?’ *Health Research Policy and Systems*, 17 (1), p. 33. doi: [10.1186/s12961-019-0432-3](https://doi.org/10.1186/s12961-019-0432-3).

Williams, O., Sarre, S., Papoulias, S. C., Knowles, S., Robert, G., Beresford, P., Rose, D., Carr, S., Kaur, M. and Palmer, V. J. (2020). ‘Lost in the shadows: reflections on the dark side of co-production’. *Health Research Policy and Systems*, 18 (1), p. 43. doi: [10.1186/s12961-020-00558-0](https://doi.org/10.1186/s12961-020-00558-0).
